# Predictive value of abbreviated olfactory tests in prodromal Parkinson disease

**DOI:** 10.1038/s41531-023-00530-z

**Published:** 2023-06-29

**Authors:** Pavan A. Vaswani, James F. Morley, Danna Jennings, Andrew Siderowf, Kenneth Marek, the PARS Investigators, John Seibyl, Matthew Stern, David Russell, Kapil Sethi, Samuel Frank, Tanya Simuni, Robert Hauser, Bernard Ravina, Irene Richards, Grace Liang, Charles Adler, Rachel Saunders-Pullman, Marian L. Evatt, Eugene Lai, Indu Subramanian, Penelope Hogarth, Kathryn Chung

**Affiliations:** 1 Parkinson’s Disease Research, Education and Clinical Center, Corporal Michael J. Crescenz VA Medical Center, Philadelphia, PA USA; 2 Department of Neurology, Perelman School of Medicine, University of Pennsylvania, Philadelphia, PA USA; 3 The Institute for Neurodegenerative Disorders, New Haven, CT USA; 4 University of Pennsylvania, Philadelphia, PA USA; 5 Medical College of Georgia, Augusta, GA USA; 6 Boston University Medical Center, Boston, MA USA; 7 Northwestern University, Chicago, Ill USA; 8 University of South Florida, Tampa, FL USA; 9 University of Rochester, Rochester, NY USA; 10 The Parkinson’s Institute, Sunnyvale, CA USA; 11 Mayo Clinic Arizona, Scottsdale, AZ USA; 12 Beth Israel Medical Center, New York, NY USA; 13 Emory University, Atlanta, GA USA; 14 Michael E. DeBakey Department of Veteran’s Affairs Medical Center, Houston, TX USA; 15 UCLA Medical Center, Los Angeles, CA USA; 16 Portland VA Medical Center, Portland, OR USA

**Keywords:** Parkinson's disease, Diagnostic markers, Risk factors

## Abstract

There is disagreement in the literature whether olfaction may show specific impairments in Parkinson Disease (PD) and if olfactory tests comprised of selected odors could be more specific for diagnosis. We sought to validate previously proposed subsets of the University of Pennsylvania Smell Identification Test (UPSIT) odors for predicting conversion to PD in an independent, prodromal cohort. Conversion to PD was assessed in 229 participants in the Parkinson At Risk Study who completed baseline olfactory testing with the UPSIT and up to 12 years of clinical and imaging evaluations. No commercially available or proposed subset performed better than the full 40-item UPSIT. The proposed “PD-specific” subsets also did not perform better than expected by chance. We did not find evidence for selective olfactory impairment in Parkinson disease. Shorter odor identification tests, including commercially available 10–12 item tests, may have utility for ease of use and cost, but not for superior predictive value.

## Introduction

Impaired olfaction is a common prodromal sign of Parkinson disease (PD), preceding the diagnosis of PD by 4 or more years^1,2^. The rate of hyposmia in clinically diagnosed PD ranges from 50% to over 90%^3–9^, depending on the population and diagnostic criteria used. Given the high prevalence and prodromal onset of olfactory impairment, screening for hyposmia has been of interest for the early identification of prodromal patients for recruitment in clinical trials of neuroprotection^10,11^. More than half of hyposmic PD patients are unaware of the deficit^3,12,13^, highlighting the need for objective assessments, and so a variety of tests have been developed for formal testing^14,15^. One of the most widely used tests is the University of Pennsylvania Smell Identification Test (UPSIT), a 40-item odor identification test which can be self-administered in ~15 min^16^.

The UPSIT was initially developed to detect hyposmia in the general population but has also been evaluated in patient groups. Several studies using the UPSIT have investigated whether there may be a pattern of selective odor detection loss in PD relating to the different underlying pathobiology. Doty et al. first noted that the olfactory impairments in PD affected all odors, and so suggested that impairments were general, not specific^3^. Subsequent work, however, has reported that some individual odors^5,17–20^ or combinations of odors^21–23^ appear to better differentiate PD patients from healthy controls, suggesting that, by using subsets of UPSIT items, shorter tests could be developed with better diagnostic characteristics for PD than the full 40-item UPSIT.

One criticism of most of these analyses is the lack of validation in an independent cohort^20^, although some did use a validation cohort or cross validation techniques^20,22,23^. In addition, many studies identified odors that have apparent greater discriminatory power in their cohorts, but did not test their subset against a null hypothesis, that is whether the odors perform better than would be expected if they were selected by chance^5,17–19,22,23^. Others have noted that a wide range of odorants have been proposed without agreement across studies^7^. The hypothesis of selective odor loss in PD has also not been assessed in a prodromal group, where improved screening would have the highest value.

In this study, we review the previously proposed subsets of odorants in the UPSIT hypothesized to be selectively impaired in PD and test their performance in a large well-characterized, independent, prodromal cohort. We compare each proposed subset to the performance of the full UPSIT and each other. In addition, we assess whether the putative “PD-specific” odor sets perform better than would be expected by chance, using two metrics that have been previously used to assess odor combinations in PD.

## Results

### Previously proposed “PD-specific” subsets

**Fig. 1:**
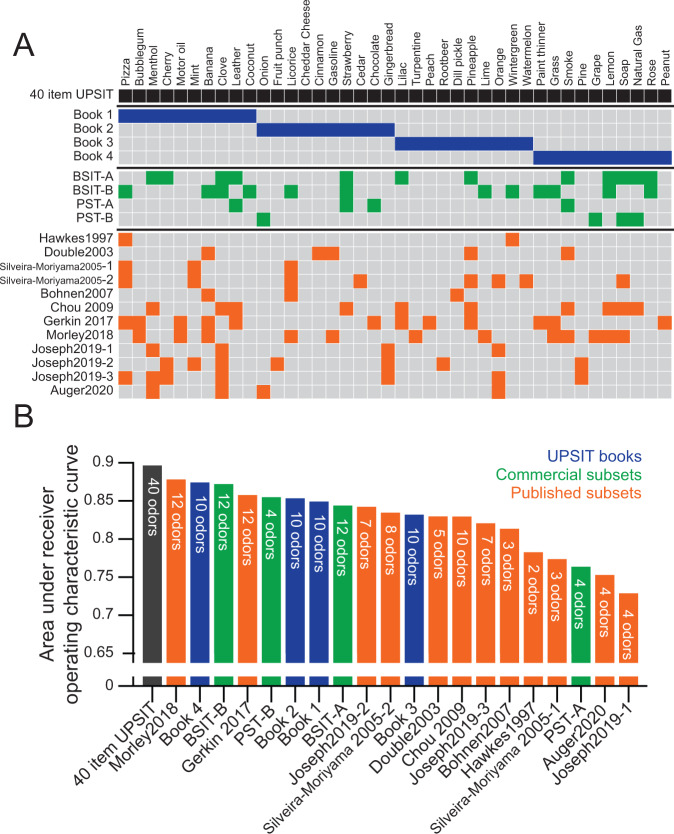
Performance of proposed and commercially available odor subsets. **A** The UPSIT consists of four 10-item books (top), which can be administered individually. Four commercial subsets of odors (middle) have been produced—two versions of the 12-item Brief Smell Identification Test (B-SIT), and two versions of the 4-item Pocket Smell Test (PST). Several publications (bottom) have evaluated other combinations of odors and, in some cases, proposed that they may have particular value in discriminating PD from healthy controls. In some cases, multiple combinations were proposed, denoted by −1, −2, or −3. Filled squares indicate odors included in the subset. **B** Discriminatory power of commercially available and proposed odor subsets. No subset outperformed the full 40-item UPSIT in this independent prodromal cohort.

A search for manuscripts using the UPSIT or B-SIT and review of these manuscripts, identified 8 manuscripts and abstracts proposing or evaluating subsets of odors for the identification of PD^5,17,18,20–24^. Several other studies captured by this search reported odors that are individually more or less effective at discriminating healthy controls from patients with PD, but did not specifically propose a combination of odors^25–34^. In these publications, the proposed subsets consisted of 2–12 odors, which were widely variable across manuscripts (Fig. 1A). No odor was included in more than half the proposed subsets; 37 of the 40 UPSIT odors were included in at least one subset.

### Performance of proposed and commercially available subsets

We evaluated the ability of the proposed subsets of odors to predict conversion to PD in this independent cohort, using two metrics: (1) the area under the receiver operating characteristic curve (AUC) (Fig. 1B); and (2) the sum of the sensitivity of and specificity (Supplemental Fig. 1A), each of which has been used in prior work^20,22,23^. Of the 229 PARS participants included in this analysis, 31 went on to develop either DAT deficit or a clinical diagnosis of PD (25 DAT deficit, 20 clinical diagnosis; of these, 14 developed both) in up to 10 years of follow-up. No proposed subset outperformed the full, 40-item UPSIT with either metric. The full UPSIT had statistically significantly greater AUC than all other combinations (*p* < 0.05), except for Book 4 (*p* = 0.26); Book 2 (*p* = 0.065); Morley^20^, a 12-item subset experimentally derived from the 40-item UPSIT (*p* = 0.35); and PST-B, a 4-item commercially available test (*p* = 0.07). In general, performance of the subsets was correlated with the number of odorants on the proposed or commercially available subsets (number of items vs AUC: *R* = 0.75, *p* < 0.001).

**Fig. 2:**
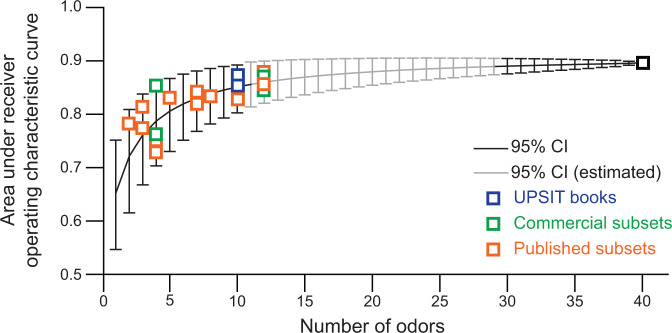
Expected performance of odor combinations selected by chance, based on length. The mean and 95% confidence interval for the expected AUC of a randomly selected combination of odors of a given length (1 odor to 40 odors) was computed (black) or estimated (gray). The performance of each proposed or commercially available subset is shown relative to these intervals (squares). Only the Pocket Smell Test Version B (PST-B, 4 items) was outside of the 95% confidence interval for length; but was within the 99% confidence interval (not shown).

We next considered whether the proposed subsets performed better than predicted by chance for their length, as would be expected if they were specific for PD. None of the published available subsets performed outside the 95% confidence interval for the performance of a random combination of odors of the same length, using either metric (Fig. 2, Supplemental Fig. 1C). Only one commercial subset, the Pocket Smell Test Version B, was outside this interval but within the 99% confidence interval. As 20 subsets were considered with each of two metrics, up to two subsets outside the 95% confidence interval would be expected by chance.

## Discussion

We demonstrate that neither previously proposed “PD-specific” odor subsets nor commercially available shorter versions of the UPSIT perform better than the full test or better than expected by chance for their length at identifying prodromal PD. Taken with the wide variability across studies in the odorants proposed to be PD-specific, these results argue against selective odor identification loss in PD.

It is important to note that these results do not indicate that testing with a shorter subset of odors does not have value for other reasons. To the contrary, we observe that discriminatory power increases dramatically with each added odor up to ~10–12 odorants; but that there is more modest added benefit for additional items (Fig. 2). Conveniently, several commercial subsets are this length: the use of one of the UPSIT books (10 items each) or B-SIT versions (12 items) is likely reasonable when there are time or cost constraints, for example, in a busy clinical practice or for screening large numbers of potential participants. However, this finding should be validated in independent prospective studies.

Other work also supports our conclusion. Boestveldt et al.^7^ also noted that the “sheer number of different odors across the various studies” argues against selective odor loss in PD. Chou et al.^21^ compared a subset of odors proposed to be selective for PD with one proposed for Alzheimer disease, and found performance on the two combinations to be correlated and both impaired in PD, and thus conclude the olfactory impairment in these conditions is likely non-specific. Morley et al.^20^ found that a variety of odors could be selected as ‘PD specific’ depending on the method used, but that these subsets did not retain discriminatory power when validated in two independent cohorts. Markopoulou et al.^35^ repeated the administration of the B-SIT in the same PD patients and controls after a year long interval. There was poor intra-rater agreement regarding the specific items that were missed on the two tests conducted one year apart by the same individual, arguing strongly against selective odor identification loss even in an individual patient. Our analysis similarly argues against selective odor loss in the population.

The lack of consistent evidence for selective odor loss is likely related to both the neurophysiology of odor detection and identification as well as limitations of abbreviated odor identification testing most commonly used to evaluate olfactory function. When an odorant is present, an array of olfactory receptor neurons are activated, each with one of ~400 odor receptors^36^. The relative activation of each receptor and cell in that array reflects an odor’s identity—no one olfactory neuron or receptor corresponds to a single odor, though one receptor can have a large role on the perception of an odorant in some cases. As such, even selective loss of neurons with a subset of receptors is unlikely to result in total loss of ability to detect a single odor, though will likely make some odors more challenging to detect than others. Similarly, despite describing an”odor” as a singular entity, most common odorants are in fact multiple chemicals, each of which partially activate a set of receptors; and conversely, multiple and distinct combinations of chemicals can be perceived as the same odor.

The limitations of clinical tests of olfaction also likely contribute to these findings. Olfaction can be tested in multiple ways: odor identification tests (e.g. the UPSIT), odor detection or recognition threshold tests (e.g. Snap and Sniff), odor discrimination tests, among others^37^. The UPSIT uses odor identification, presenting 40 odorants with a multiple choice, forced-choice format. Each item is scored as correct or incorrect—and so this test does not differentiate moderate dysfunction from more severe difficulties in identifying a single odorant, though can assess degrees of olfactory dysfunction overall. Furthermore, an incorrect answer could be due to difficulty detecting and identifying the odorant or, separately, difficulty discriminating from the particular incorrect choices presented. Scoring these tests also requires selection of a cutoff point to be considered normosmic; shorter tests will necessarily have more granular scoring and thus potentially be less sensitive and more prone to misclassification of patients with or without olfactory dysfunction. Cultural issues may also contribute: some odors (or distractor choices) may have greater familiarity or salience to some individuals. Fatigue, attention, and cognitive impairments, which are likely more impaired in people with PD, could also negatively affect performance. Even in healthy adults, there are polymorphisms in odorant receptor genes that can affect odor identification^36^. Taken together, differences in cultural familiarity, interindividual variability, and granular nature of scoring likely make shorter tests mask more subtle population differences, if present.

There are several limitations of this work. Prior studies identified and proposed odor subsets in clinically diagnosed PD patients, while this analysis is in a prodromal cohort. It has been hypothesized that synuclein aggregates first appear in the olfactory bulb and dorsal motor nucleus of the vagus (“body-first” prodromal PD), hence the interest in hyposmia as a prodromal feature of PD. However, more recent data has suggested that there may be “brain-first” prodromal PD where the olfactory bulb is less affected in early disease^38^. Prodromal patients may have less, more variable, or different degrees of olfactory bulb involvement than clinically diagnosed or advanced patients. However, if and when the olfactory bulb is involved, our expectation would be that any “PD-specific” odors to also be specific in the prodromal phase. Furthermore, as olfaction is of particular interest in screening and identifying early and prodromal patients for trials of potential disease modifying therapies, evaluation of the hypothesis that there are ‘PD specific’ odors is likely of greatest interest in the early/prodromal stage.

Our evaluation of odor subsets was completed “in silico”, similar to prior studies, which does not account for potential effects of the order of presentation of odors or fatigue which would be different in a shorter test. Direct evaluation of odor subsets could be used to mitigate this limitation, but would be challenging given the number of permutations of odor subsets that can be created. In our cohort, the first odors tested, “Book1”, of the UPSIT did not show superior performance as compared to the subsequent books, which suggests that the effects of fatigue and order may be small.

In summary, we did not find evidence of “PD-specific” odors in this large, prodromal cohort. The use of abbreviated, 10–12 item smell tests is likely reasonable from the perspective of time, cost, and ease of use, but is unlikely to have added or specific discriminatory power in identification of prodromal PD.

## Methods

The recruitment and assessments in the Parkinson Associated Risk Syndrome (PARS) study have been previously described^39^. The PARS study was approved by the Western Institutional Review Board, the Human Research Protection Office at the US Army Medical Research Material and Command, and the local institutional review boards at each center. The Clinicaltrials.gov registry number is NCT00387075. Briefly, participants without evidence of parkinsonism on initial exam or known conditions that affect olfaction were recruited from online outreach, mailing lists, and relatives of patients with PD and provided written informed consent. Olfaction was assessed at baseline with the UPSIT, which was mailed to participants and self-administered. The UPSIT is a 40-item smell test consisting of four 10-item books. Each page contains one microencapsulated (‘scratch and sniff’) odorant and four choices for the identification of the odorant. A choice must be provided for each odorant. A total of 4999 participants completed and returned questionnaires, and 669 of these participants were hyposmic (olfaction less than the 15th percentile for age and gender). Hyposmic participants were invited to participate in the longitudinal study; 203 hyposmic participants and 100 age and sex matched normosmic participants were followed in this study. Three participants were excluded as they were taking modafinil at the time of the baseline DAT scan. The responses for individual odors were recorded for initial olfactory assessment for 229 participants in PARS (134 hyposmic, 95 normosmic) and included in this analysis. Baseline characteristics for the hyposmic and normosmic cohorts are shown in Table 1.

**Table 1:** Baseline characteristics of the participants in the PARS study where performance on individual smells was recorded.

		Hyposmic at baseline (*n* = 134)	Normosmic at baseline (*n* = 95)
Age (years)		62.4 (8.2)	62.3 (10.4)
Male sex (*n*, %)		53 (39.6%)	42 (44.2%)
First degree relative with PD (*n*, %)		70 (52.2%)	48 (50.5%)
Baseline olfaction (age adjusted %ile)		6.6 (4.1)	70.5 (32.1)
Baseline DAT (% age-expected lowest putamen specific binding ratio)		91.9 (22.2)	101.5 (15.3)
Conversion to PD	Clinical	20	0
	Imaging	25	0
	Either clinical or imaging	31	0

Hyposmic and age and gender matched normosmic participants were followed longitudinally. Data are mean (SD) except where noted.

Conversion to PD was assessed with annual clinical evaluations and [^123^I] β-CIT SPECT (DAT) scans at baseline and approximately every 2 years thereafter. Conversion to PD was defined as clinical diagnosis on annual assessments or, alternatively, evidence of a DAT deficit (i.e. a putamen specific binding ratio <65% of that expected for healthy peers of the same age), at any point in the up to 10 years of clinical and imaging follow-up. DAT deficit using this criterion has been shown to predict a high rate of conversion to clinical diagnosis^40^.

### Proposed odor subsets

A PubMed search was conducted with search terms [“University of Pennsylvania Smell Identification Test” or “Brief Smell Identification Test”], and [“Parkinson disease” or “Parkinson’s disease”] to identify potential manuscripts that proposed or evaluated subsets of odors of the UPSIT for the identification of PD. The search was limited to English language manuscripts. This search yielded 140 results; of these eight manuscripts proposed or evaluated subsets of odors for the identification of PD^5,17,20–24^. Review of these manuscripts identified an additional abstract^18^.

The ability of proposed odor subsets and commercially available tests to predict conversion to PD was calculated using the area under the receiver operating characteristic curve (AUC). Nonparametric Mann–Whitney generalized U statistics accounting for covariance were used to compare AUCs^41^. The greatest sum of the sensitivity and specificity at the best threshold for each subset was also separately evaluated as a metric of performance, because this has been used in prior work and proposed to provide distinct results from AUC^22,23^. Linear regression was used to compare the association between the two metrics.

### Expected performance of odor subsets

Next, in order to test the proposed odor subsets against a null hypothesis, we compared the performance of proposed and commercially available subsets to the distribution of performance of randomly selected subsets of the same length (number of odorants), to evaluate if the proposed and commercial subsets of putative ‘PD specific’ odors performed better than expected if the specific odorants were chosen by chance.

To compute the distribution of performance of subsets with 10 or fewer odors or 30 or more odors, the performance of all combinations of odors of that length was computed with each metric (AUC and best sum of sensitivity and specificity). For subsets of length >10 and <30, the performance of 1 million randomly selected subsets of each length was computed, as the computational time to examine all possibilities for these lengths was prohibitive (i.e. there are 847,660,528 subsets of 10 items; but 137,846,528,820 subsets of 20 items). The proposed and commercial subsets’ performance were then compared to this distribution.

Analyses were completed using MATLAB (R2015a). Mann–Whitney *U* statistics were calculated using StAR^41^.

### Reporting summary

Further information on research design is available in the Nature Portfolio Reporting Summary linked to this article.

## Supplementary information

Supplementary Material

Nature reporting summary checklist

## Data Availability

Data that support the findings of this study are available from the corresponding author upon request.
