# Supplementary material for: Predictive value of abbreviated olfactory tests in prodromal Parkinson disease
Source: NPJ Parkinsons Dis. 2023 Jun 29;9:103. doi: 10.1038/s41531-023-00530-z (PMC10310835; doi:10.1038/s41531-023-00530-z)
Supplement: Supplementary file 1 — Supplementary Material [file 41531_2023_530_MOESM1_ESM.pdf]

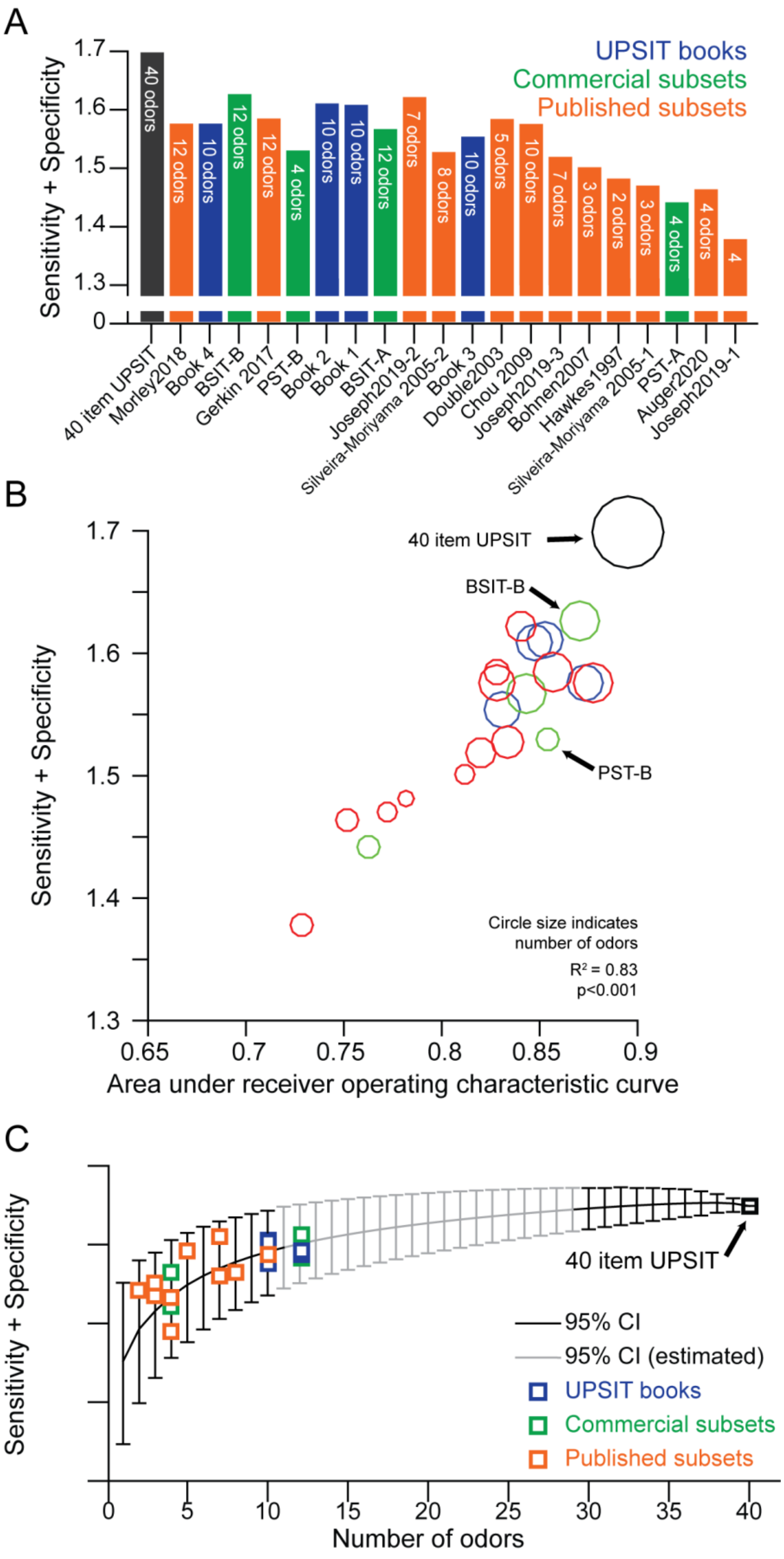
A
Sensitivity + Specificity
UPSIT books
Commercial subsets
Published subsets
40 odors
12 odors
10 odors
12 odors
12 odors
4 odors
10 odors
10 odors
12 odors
7 odors
8 odors
10 odors
5 odors
10 odors
7 odors
3 odors
2 odors
3 odors
4 odors
4 odors
4
40 item UPSIT
Morley2018
Book 4
BSIT-B
Gerkin 2017
PST-B
Book 2
Book 1
BSIT-A
Joseph2019-2
Silveira-Moriyama 2005-2
Book 3
Double2003
Chou 2009
Joseph2019-3
Bohnen2007
Hawkes1997
Silveira-Moriyama 2005-1
PST-A
Auger2020
Joseph2019-1
B
40 item UPSIT
BSIT-B
PST-B
Circle size indicates
number of odors
R² = 0.83
p<0.001
Sensitivity + Specificity
Area under receiver operating characteristic curve
C
Sensitivity + Specificity
40 item UPSIT
95% CI
95% CI (estimated)
UPSIT books
Commercial subsets
Published subsets
Number of odors

Supplemental Figure 1: Performance of proposed and commercial subsets using sum of sensitivity and specificity at best threshold metric. (A) Performance of commercially available and proposed odor subsets was also computed using the sum of the sensitivity and specificity at the best threshold for that odor combination in this cohort. No combination outperformed the full 40-item UPSIT. The order of the x-axis is the same as in Figure 1B. (B) The two metrics (AUC and sensitivity + specificity) were correlated ($R^2 = 0.83$, $p<0.001$). Each circle represents one proposed or commercially available combination in Figure 1A; the area of each circle is proportional to the number of items in that combination. (C) The distribution of this metric was computed (black) or estimated (gray) for each combination of odors of a given length. The mean and 95% confidence interval are shown. No proposed or commercially available subsets (black squares) were outside the 95% confidence interval for the performance of a randomly selected subset of the same length
