# Supplementary material for: Predictive value of abbreviated olfactory tests in prodromal Parkinson disease
Source: NPJ Parkinsons Dis. 2023 Jun 29;9:103. doi: 10.1038/s41531-023-00530-z (PMC10310835; doi:10.1038/s41531-023-00530-z)
Supplement: Supplementary file 2 — Nature reporting summary checklist [file 41531_2023_530_MOESM2_ESM.pdf]

nature portfolio

Corresponding author(s): Pavan Vaswani MD PhD

Last updated by author(s): 2023/05/09

Reporting Summary

Nature Portfolio wishes to improve the reproducibility of the work that we publish. This form provides structure for consistency and transparency in reporting. For further information on Nature Portfolio policies, see our Editorial Policies and the Editorial Policy Checklist.

Statistics

For all statistical analyses, confirm that the following items are present in the figure legend, table legend, main text, or Methods section.

n/a	Confirmed	
☐	☒	The exact sample size (*n*) for each experimental group/condition, given as a discrete number and unit of measurement
☐	☒	A statement on whether measurements were taken from distinct samples or whether the same sample was measured repeatedly
☐	☒	The statistical test(s) used AND whether they are one- or two-sided<br>*Only common tests should be described solely by name; describe more complex techniques in the Methods section.*
☒	☐	A description of all covariates tested
☐	☒	A description of any assumptions or corrections, such as tests of normality and adjustment for multiple comparisons
☐	☒	A full description of the statistical parameters including central tendency (e.g. means) or other basic estimates (e.g. regression coefficient) AND variation (e.g. standard deviation) or associated estimates of uncertainty (e.g. confidence intervals)
☐	☒	For null hypothesis testing, the test statistic (e.g. *F*, *t*, *r*) with confidence intervals, effect sizes, degrees of freedom and *P* value noted<br>*Give P values as exact values whenever suitable.*
☒	☐	For Bayesian analysis, information on the choice of priors and Markov chain Monte Carlo settings
☒	☐	For hierarchical and complex designs, identification of the appropriate level for tests and full reporting of outcomes
☐	☒	Estimates of effect sizes (e.g. Cohen's *d*, Pearson's *r*), indicating how they were calculated

Our web collection on statistics for biologists contains articles on many of the points above.

Software and code

Policy information about availability of computer code

Data collection	n/a
Data analysis	Analyses were completed using MATLAB (R2015a). MannWhitney U statistics were calculated using StAR (reference provided)

For manuscripts utilizing custom algorithms or software that are central to the research but not yet described in published literature, software must be made available to editors and reviewers. We strongly encourage code deposition in a community repository (e.g. GitHub). See the Nature Portfolio guidelines for submitting code & software for further information.

Data

Policy information about availability of data

All manuscripts must include a data availability statement. This statement should provide the following information, where applicable:

- Accession codes, unique identifiers, or web links for publicly available datasets
- A description of any restrictions on data availability
- For clinical datasets or third party data, please ensure that the statement adheres to our policy

Data that support the findings of this study are available from the corresponding author upon request

Research involving human participants, their data, or biological material

Policy information about studies with human participants or human data. See also policy information about sex, gender (identity/presentation), and sexual orientation and race, ethnicity and racism.

Reporting on sex and gender	Sex was collected by self-report. Disaggregated data is presented
Reporting on race, ethnicity, or other socially relevant groupings	Not used
Population characteristics	Presented in Table 1
Recruitment	Presented in methods
Ethics oversight	The PARS study was approved by the Western Institutional Review Board, the Human Research Protection Office at the US Army Medical Research Material and Command, and the local institutional review boards at each center. The Clinicaltrials.gov registry number is NCT00387075.

Note that full information on the approval of the study protocol must also be provided in the manuscript.

Field-specific reporting

Please select the one below that is the best fit for your research. If you are not sure, read the appropriate sections before making your selection.

☒ Life sciences ☐ Behavioural & social sciences ☐ Ecological, evolutionary & environmental sciences

For a reference copy of the document with all sections, see nature.com/documents/nr-reporting-summary-flat.pdf

Life sciences study design

All studies must disclose on these points even when the disclosure is negative.

Sample size	Available PARS data was used. Hyposmic participants willing to participate in the PARS longitudinal study and 100 age and sex matched normosmic controls were enrolled in the study. Three participants were excluded as they were taking modafinil at the time of the baseline DAT scan. The responses for individual odors were recorded for initial olfactory assessment for 229 participants in PARS (134 hyposmic, 95 normosmic) and included in this analysis.
Data exclusions	Three participants were excluded as they were taking modafinil at the time of the baseline DAT scan, which can affect its interpretation.
Replication	Performance of olfactory subsets was compared to randomly selected subsets of the same number of odorants.
Randomization	No randomization of participants was performed
Blinding	No blinding was performed as no treatments were administered

Reporting for specific materials, systems and methods

We require information from authors about some types of materials, experimental systems and methods used in many studies. Here, indicate whether each material, system or method listed is relevant to your study. If you are not sure if a list item applies to your research, read the appropriate section before selecting a response.

Materials & experimental systems

n/a	Involved in the study	
☒	☐	Antibodies
☒	☐	Eukaryotic cell lines
☒	☐	Palaeontology and archaeology
☒	☐	Animals and other organisms
☐	☒	Clinical data
☒	☐	Dual use research of concern
☒	☐	Plants

Methods

n/a	Involved in the study	
☒	☐	ChIP-seq
☒	☐	Flow cytometry
☒	☐	MRI-based neuroimaging

Clinical data

Policy information about clinical studies

All manuscripts should comply with the ICMJE guidelines for publication of clinical research and a completed CONSORT checklist must be included with all submissions.

Clinical trial registration	NCT00387075
Study protocol	https://clinicaltrials.gov/ct2/show/NCT00387075, http://www.parsinfosource.com/
Data collection	Beth Israel Medical Center - New York, NY<br>Boston University School of Medicine - Boston, MA<br>Emory University School of Medicine - Atlanta, GA<br>Institute for Neurodegenerative Disorders - New Haven, CT<br>Mayo Clinic Scottsdale - Scottsdale, AZ<br>Medical College of Georgia - Augusta, Ga<br>Michael E. Debakery VA Medical Center - Houston, TX<br>Northwestern University - Chicago, IL<br>Pennsylvania Hospital PDMDC - Philadelphia, PA<br>The Parkinson Institute - Sunnyvale, CA<br>UCLA/SW PADRECC - Los Angeles, CA<br>University of North Carolina - Chapel Hill, NC<br>University of Rochester - Rochester, NY<br>University of South Florida PDMDC - Tampa, FL<br>Portland VA Medical Center - Portland, OR
Outcomes	Clinical assessments and DAT scans as described in Methods
